# The Flow-Induced Degradation and Vascular Cellular Response Study of Magnesium-Based Materials

**DOI:** 10.3389/fbioe.2022.940172

**Published:** 2022-07-07

**Authors:** Tengda Shang, Kebing Wang, Shusheng Tang, Yang Shen, Lei Zhou, Lu Zhang, Yuancong Zhao, Xin Li, Lin Cai, Jin Wang

**Affiliations:** ^1^ School of Materials Science and Engineering, Southwest Jiaotong University, Chengdu, China; ^2^ Department of Cardiology, Third People’s Hospital of Chengdu Affiliated to Southwest Jiaotong University, Chengdu, China

**Keywords:** magnesium-based materials, flow-induced degradation, biodegradable stent, shear stress, vascular cellular responses

## Abstract

Magnesium (Mg)-based materials are considered as potential materials for biodegradable vascular stents, and some Mg-based stents have obtained regulatory approval. However, the development and application of Mg-based stents are still restricted by the rapid degradation rate of Mg and its alloys. In order to screen out the desirable Mg-based materials for stents, the degradation behavior still needs further systematic study, especially the degradation behavior under the action of near-physiological fluid. Currently, the commonly used Mg-based vascular stent materials include pure Mg, AZ31, and WE43. In this study, we systematically evaluated their corrosion behaviors in a dynamic environment and studied the effect of their degradation products on the behavior of vascular cells. The results revealed that the corrosion rate of different Mg-based materials was related to the composition of the elements. The dynamic environment accelerated the corrosion of Mg-based materials. All the same, AZ31 still shows good corrosion resistance. The effect of corrosive products on vascular cells was beneficial to re-endothelialization and inhibition of smooth muscle cell proliferation at the implantation site of vascular stent materials.

## Introduction

Coronary artery disease (CAD), which has one of the highest incidences and mortality rates, has become a worldwide public health problem (He et al., 2019). Vascular stent implantation in the site of vascular lesions can support diseased narrow blood vessels to ensure smooth blood flow (Erbel et al., 2007). Due to its small trauma and high efficiency, the interventional treatment of vascular stents has become an important method for the treatment of CAD (Hermawan et al., 2010; Pant et al., 2011). However, the problems of the nonbiodegradable stent, such as late thrombosis, inflammation, and restenosis in stents, often lead to the implantation failure (Fu et al., 2020). In recent years, the biodegradable stent made from the biodegradable polymers and metals has attracted more and more attention, thanks to their acceptable biodegradability and biocompatibility (Jinnouchi et al., 2019; Zhou et al., 2019). Biodegradable stents can be absorbed and metabolized by the human body after the service time. In this way, the second operation can be avoided, the pain and economic burden of patients can be alleviated (Kalb et al., 2012; Dziuba et al., 2013). Owing to their superior mechanical strength and toughness compared to the biodegradable materials such as polymers, biodegradable metal materials are more suitable for load-bearing applications (Yang et al., 2018).

Magnesium (Mg) and its alloys are promising materials for biodegradable vascular stents (Oliver et al., 2021; Zhang et al., 2021). Research in recent years has shown that Mg^2+^ has strong anti-inflammatory effect. Mg^2+^ can improve lipid profile, reduce free oxygen radicals, and improve endothelial function (Baaij et al., 2015). Moreover, Mg^2+^ reduce the susceptibility of coronary artery disease and have good blood compatibility (Grober et al., 2015). Magmaris developed by the Biotronik company has obtained the CE approval (Cerrato et al., 2019). Therefore, Mg-based materials are considered to be very competitive candidates for cardiovascular stent applications (Bornapour et al., 2013). However, the rapid degradation *in vivo* still limits its clinical application. Alloying is one method to improve the corrosion resistance of pure Mg (Sezer et al., 2018). Generally, Mg alloys contain aluminum (Al) or the rare earth elements (REEs) (Li et al., 2008). Alloying with Al and REEs enhances the corrosion resistance of Mg (Li et al., 2016; Erişen et al., 2022). Mg-based materials, currently under investigation as implant materials, mostly contain pure Mg, Mg–Al alloys, Mg–rare earth alloys, and so on (Wu et al., 2012; Liu et al., 2015; Törne et al., 2017; Han et al., 2019). Li et al. (2012) studied the degradation behavior of pure magnesium and WE43 under static and flowing conditions, and the results revealed that flowing conditions promoted local corrosion and increased the corrosion rate by 3–6 times. Kandala et al. (2021) reported that the degradation behavior and histological behavior of AZ31 stent implantation in the pig arteries for 28 days. The results showed that the stent remained almost intact, partially embedded in the vascular wall, and histological evidence showed no life-threatening effects. However, systematic studies on the degradation behavior and biocompatibility of these Mg-based materials are rarely reported. Therefore, we conducted a comparative study on them in this study.

The corrosion of Mg-based materials in simulated physiological environment has been extensively studied (Xin et al., 2011; Zheng et al., 2014; Han et al., 2018). Conventional evaluation methods for magnesium alloy such as immersion and hydrogen evolution are performed under static condition. However, it is well-known that stents are affected by blood flow after implantation. Hydrodynamic condition has a significant impact on the degradation of absorbable metallic stents, including degradation kinetics, degradation modes, and local pH changes (Lévesque et al., 2008; Esmaily et al., 2017). The local surface chemical changes under hydrodynamic condition with time may be different from that in the static condition. This, as a result, in turn affects corrosion and biological reactions (Robotti et al., 2014; Chen et al., 2018). Blood flow plays an important role in the initial stage of stent corrosion, because circulating blood directly contacts with the stent surface before re-endothelialization, which affects the transfer of mass such as corrosion products and hydroxide ions (Wang et al., 2016; Wang et al., 2017). It has been demonstrated that the corrosion rate of Mg-based materials rose under dynamic condition compared to static immersion (Wang et al., 2014; Liu et al., 2018). In addition to the corrosion rate, the corrosion morphology is also affected by the flow-induced shear stress (Chu et al., 2016; Gu et al., 2018). Based on the abovementioned research, it is necessary to study the degradation behavior of Mg-based materials in the flow environment. Therefore, we constructed a parallel plate flow chamber simulating the near-physiological fluid environment *in vivo*, to systematically study the flow-induced dynamic degradation behaviors of the Mg-based materials. Meanwhile, the flow-induced degradation behaviors of pure Mg, AZ31, and WE43 were compared to the degradation behaviors in traditional static immersion test. In addition, the effect of degradation products on vascular cellular behavior was also investigated.

## Materials and Methods

### Preparation of Materials

The pure Mg (99.99 wt%), AZ31 (Al 3.1 wt%, Zn 0.8 wt%, Mn 0.4 wt%), and WE43 (Y 4.3 wt%, Nd 3.0 wt%, Zr 0.5 wt%) ingots were purchased from Litmat Manufacture ChongZhou Co., Ltd., China. The Mg-based materials were cut into specimens, with 10 mm diameter and 1.5 mm thickness for static immersion experiment, with 20 mm length, 10 mm width, and 2 mm thickness for dynamic degradation experiment. The specimens were polished with SiC paper to 5000 grit, and then were cleaned ultrasonically in acetone, absolute ethanol, and distilled water to remove residues. The corrosion resistance tests were carried out in Hank’s solution (pH = 7.4 ± 0.2), which contains NaCl (8 g/L), CaCl_2_ (0.14 g/L), KCl (0.4 g/L), MgCl_2_·6H_2_O (0.1 g/L), MgSO_4_·7H_2_O (0.1 g/L), NaHCO_3_ (0.35 g/L), Na_2_HPO_4_ (0.06 g/L), KH_2_PO_4_ (0.06 g/L), and glucose (1.0 g/L).

### Static Immersion Experiment

The static corrosion behavior of the Mg-based materials was performed in Hank’s solution at 37 ± 0.5°C, which included the AZ31 group (*n* = 4), WE43 group (*n* = 4), and pure Mg group (*n* = 4), and the ratio of solution volume to surface area was 40 ml/cm^2^ according to ASTM-G31–72 standard (ASTM Standard, 2004). The Hank’s solution was refreshed every 2 days to mimic the metabolism *in vivo*. During the immersion test, the pH value was recorded by PHS-3C pH meter (Lei-ci, China), and the Mg^2+^ concentration was inspected by using an atomic absorption spectrophotometer (AAS, PERSEE, China). After different immersion periods, the samples were removed from Hank’s solution, gently rinsed with ultrapure water, and dried at room temperature. The corrosion products were removed with chromic acid solution (200 g of CrO_3_ and 10 g of AgNO_3_ per liter of water). The corrosion rate of the Mg-based materials was calculated using the following equation:

$$CR=\frac{\Delta m}{At},$$

where CR (g/m^2^/d) is the corrosion rate, ∆m (g) is the mass loss of the sample, A (m^2^) is the exposure area of the sample, and t (d) is the immersion time.

### Electrochemical Experiment

The electrochemical corrosion tests included potentiodynamic polarization (PDP) and electrochemical impedance spectroscopy (EIS), which were carried out on an electrochemical workstation (IM6, Zahner, Germany). A three-electrode system was adopted, which included a reference electrode (saturated calomel electrode, SCE), a counter electrode (platinum sheet), and the working electrode of the specimens. The exposed aera to the solution was 0.785 cm^2^. All the measurements were conducted in Hank’s solution at 37 ± 0.5°C. For the PDP test, the polarization curves were obtained from −1.8 to −0.8 V_SCE_ with a scanning rate of 1 mV/s. The corrosion potential (E_corr_) and corrosion current density (i_corr_) were determined *via* Tafel extrapolation. The cathodic Tafel slope is reliable for the Mg-based materials to obtain i_corr_ (Shi et al., 2010). The EIS scanning test was implemented at an open-circuit potential that was superposed with a sinusoidal perturbation signal of 20 mV in a frequency range of 100 kHz to 10^−2^ Hz. The EIS plots were fitted using an equivalent electrical circuit (EEC). All the electrochemical tests were repeated more than three times for the statistical relevance.

### Dynamic Degradation Experiments

**FIGURE 1:**
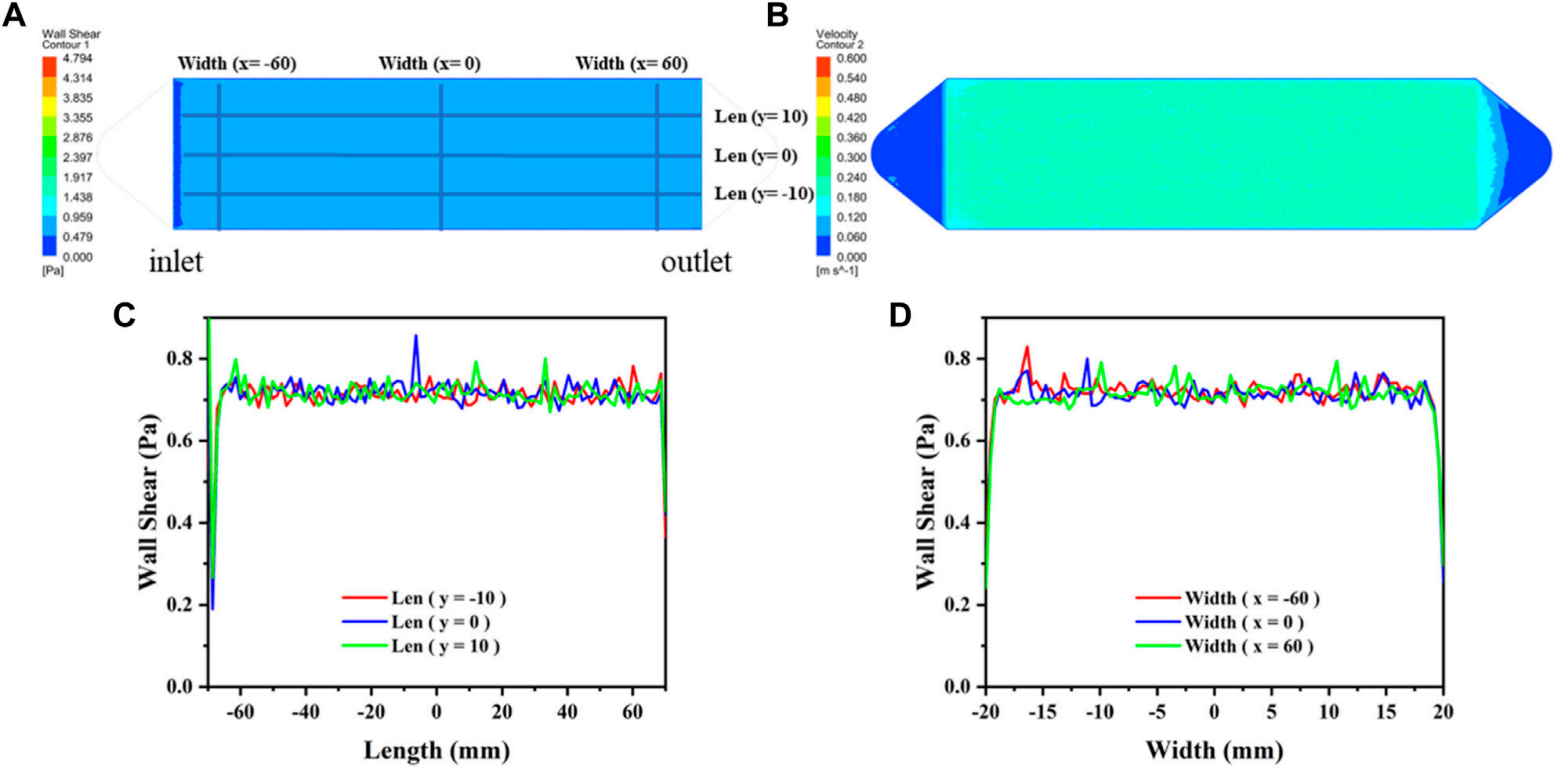
The distribution diagram of WSS and velocity in the flow chamber. **(A)** WSS distribution in the effective region. **(B)** Flow velocity distribution in the effective region. **(C)** Distribution of WSS on the length axes shown in **(A)**. **(D)** Distribution of WSS on the width axes shown in **(A)**.

To simulate the near-physiological fluid environment, a parallel plate flow chamber, which has a flow section of 4 cm in width, 0.1 cm in height, and 14 cm in length, shown in Figure 1, was designed to provide laminar flow on the surface of the samples. The mean shear stress on the surface of the samples was 0.68 Pa which is the mean wall shear stress (WSS) of the human coronary artery according to Doriot’s work (Doriot et al., 2000). The mean shear stress (τ) can be calculated as follows (Pope and Pope, 2000):

$$\tau =\frac{6\mu Q}{wh^{2}},$$

where Q is the volumetric flow rate, w and h are the width and height of the flow channel. μ is the dynamic viscosity of the Hank’s solution, which is 0.78 mPa s in this study.

**FIGURE 2:**
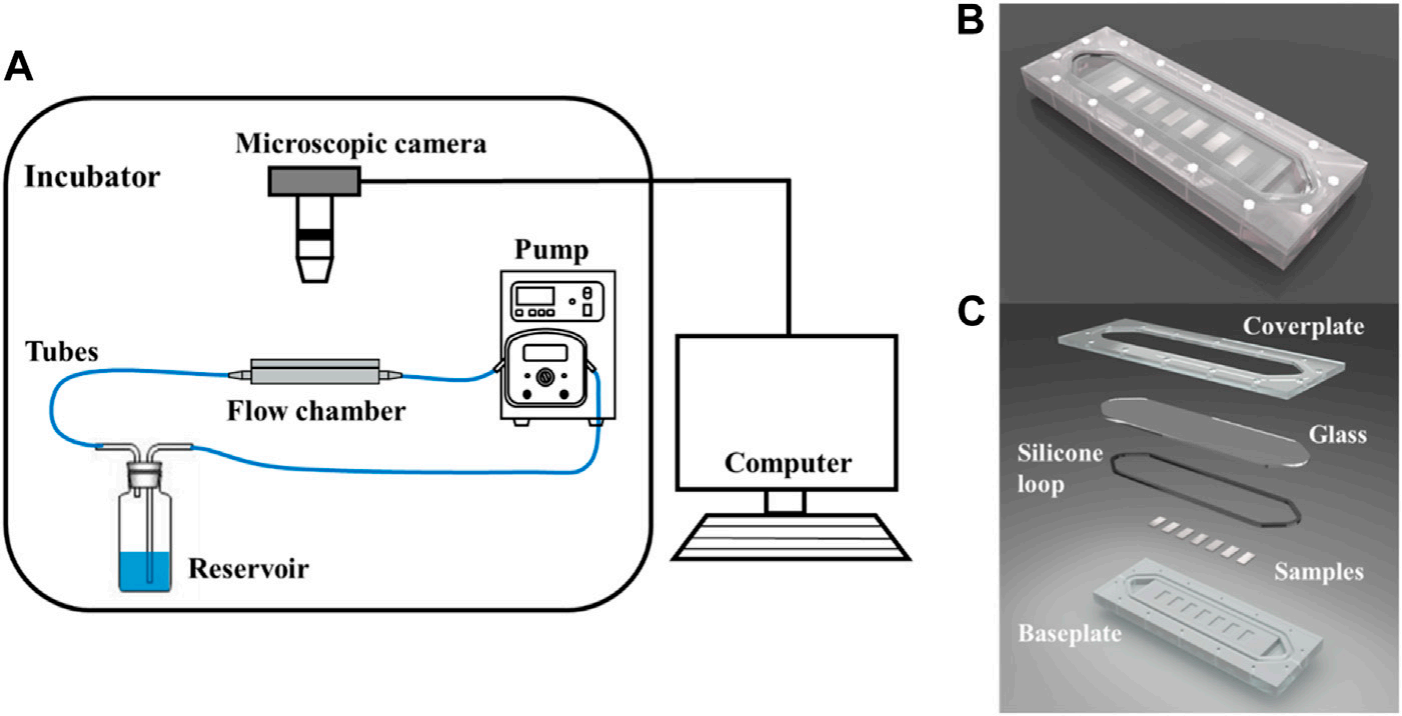
Schematic of the dynamic corrosion test devices. **(A)** Diagram of the dynamic corrosion test devices. **(B)** The macro photograph of the flow chamber. **(C)** Components of the flow chamber. The Mg-based samples (20 × 10 × 2 mm after mechanical polishing) were embedded in the parallel plate flow chamber.

The flow velocity distribution and shear stress distribution of the flow section in the flow chamber are shown in Figure 2, which was analyzed using the computational fluid dynamics (CFD) simulations *via* ANSYS FLUENT. The CFD simulations show that the mean shear stress on the sample surface is 0.68 Pa. The circulating system contains a reservoir, a peristaltic pump, a parallel plate flow chamber, several tubes, and microscopic camera with a control unit (PC computer). The surface morphology of the Mg-based samples in the flow chamber was observed and recorded using a microscopic camera. This system was placed in an incubator with a temperature of 37 ± 0.5°C. Figure 2 shows the schematic diagram of the dynamic corrosion test devices. As depicted in Figure 2C, the Mg-based samples were embedded in a parallel plate flow chamber and connected to the dynamic corrosion systems (Figure 2A). Then, the samples were exposed to a steady laminar flow for 7d respectively. After different corrosion periods, the corrosion morphologies, the pH value, and the Mg^2+^ concentration were also examined every day.

### Vascular Cellular Responses

The samples were sterilized by ultraviolet-radiation for 1 h before the preparation of extracts. Afterward, the samples were immersed in Dulbecco’s Modified Eagle’s Medium (DMEM, Gibco) supplemented with 10% fetal bovine serum (FBS, Gibco) at cell culture conditions (5% CO_2_, 95% humidity, 37 °C) for three days with a ratio of surface area to solution volume at 1.25 cm^2^/ml according to the ISO 10993-5 (International Standardization, 2009). Then, the extracts were collected without filtration.

The HUVECs and HUASMCs were acquired from the newborn umbilical veins to evaluate the vascular cellular responses of the extracts. The HUVECs and HUASMCs were cultured in DMEM supplemented with 10% FBS in the incubator (5% CO_2_, 95% humidity, 37°C). After reaching about 90% confluence, the cells were harvested with 0.25% trypsin (Sigma, United States). The digested cells were subsequently transferred to a 48-well plate with 1 × 10^4^ cells per well and precultured in DMEM (containing 10% FBS) for 24 h. Thereafter, the cell culture media were carefully replaced by the extracts of samples prepared as described previously. After 1, 3 days culture, the cells were stained by AO/PI for 8 min in a dark room. Afterward, the morphology of cells was observed by fluorescence microscopy (Olympus IX51, Japan), and the dead cells were stained red.

### Statistical Analysis

Statistical analyses were performed using a one-way analysis of variance (ANOVA). The least significant difference (LSD) test was used to determine the statistical differences between all groups. Differences were considered significant with a value of *p* < 0.05.

## Results and Discussion

### Static Corrosion Evaluation

**FIGURE 3:**
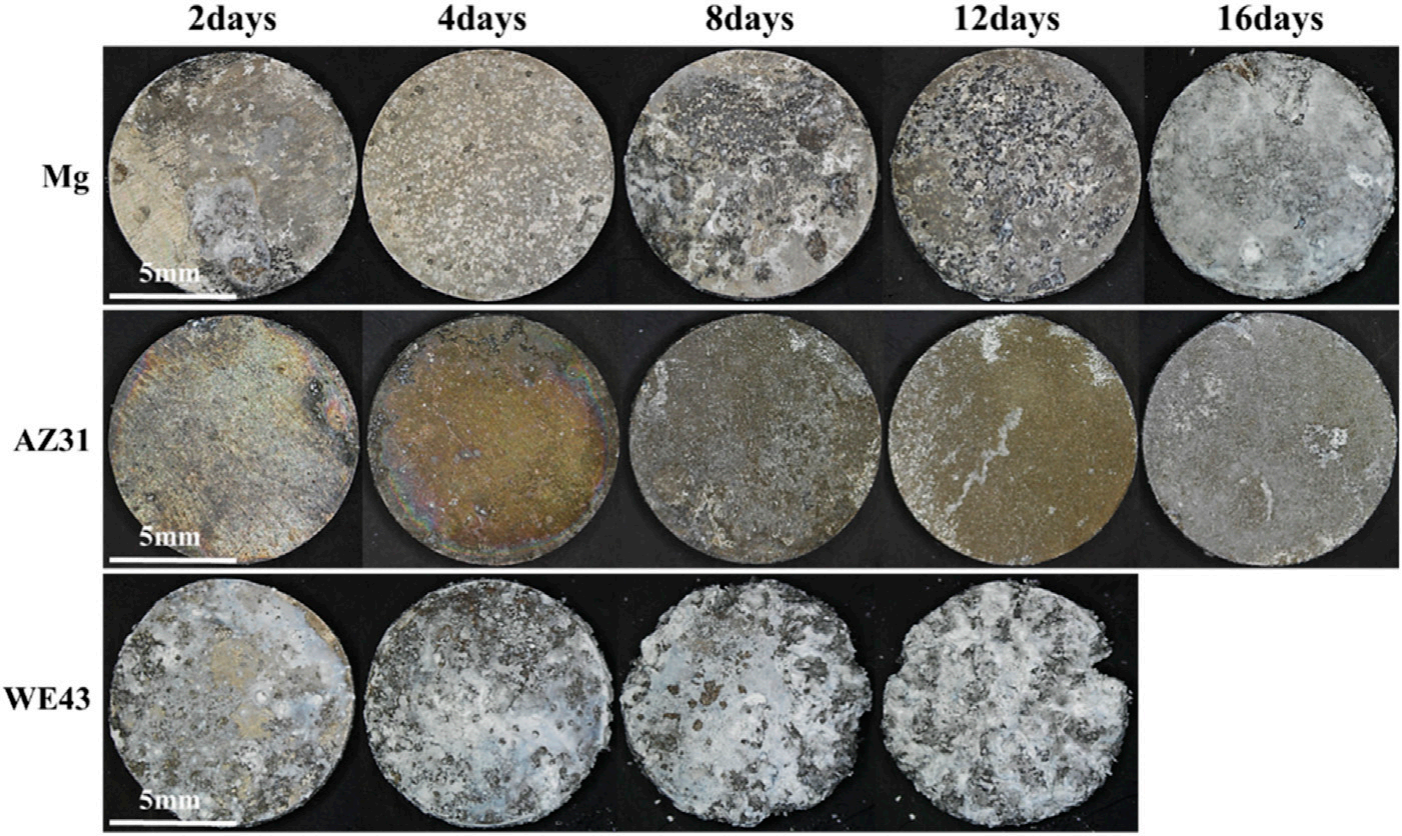
Morphologies image of Mg, AZ31, and WE43 after static immersion corrosion in Hank’s solution at 37 ± 0.5°C.

After 16 days of static immersion corrosion, Figure 3 shows that the corrosion products on the sample surface increase with time. In the pure magnesium group, there were no obvious corrosion defects and corrosion products on the sample surface after soaking for four days. After soaking for eight and 12 days, there were obvious corrosion pits and a few corrosion products on the sample surface. After soaking for 16 days, the sample surface was basically covered by corrosion products. However, the AZ31 group showed no obvious corrosion defects and corrosion products after soaking for 12 days, and was covered by a small amount of corrosion products after soaking for 16 days. The surface of WE43 group was basically covered with corrosion products after soaking for two days. WE43 had edge defect after eight days and was completely corroded on the 16th day. Compared with Mg and WE43, AZ31 had minimal morphological changes with the least corrosion products.

**FIGURE 4:**
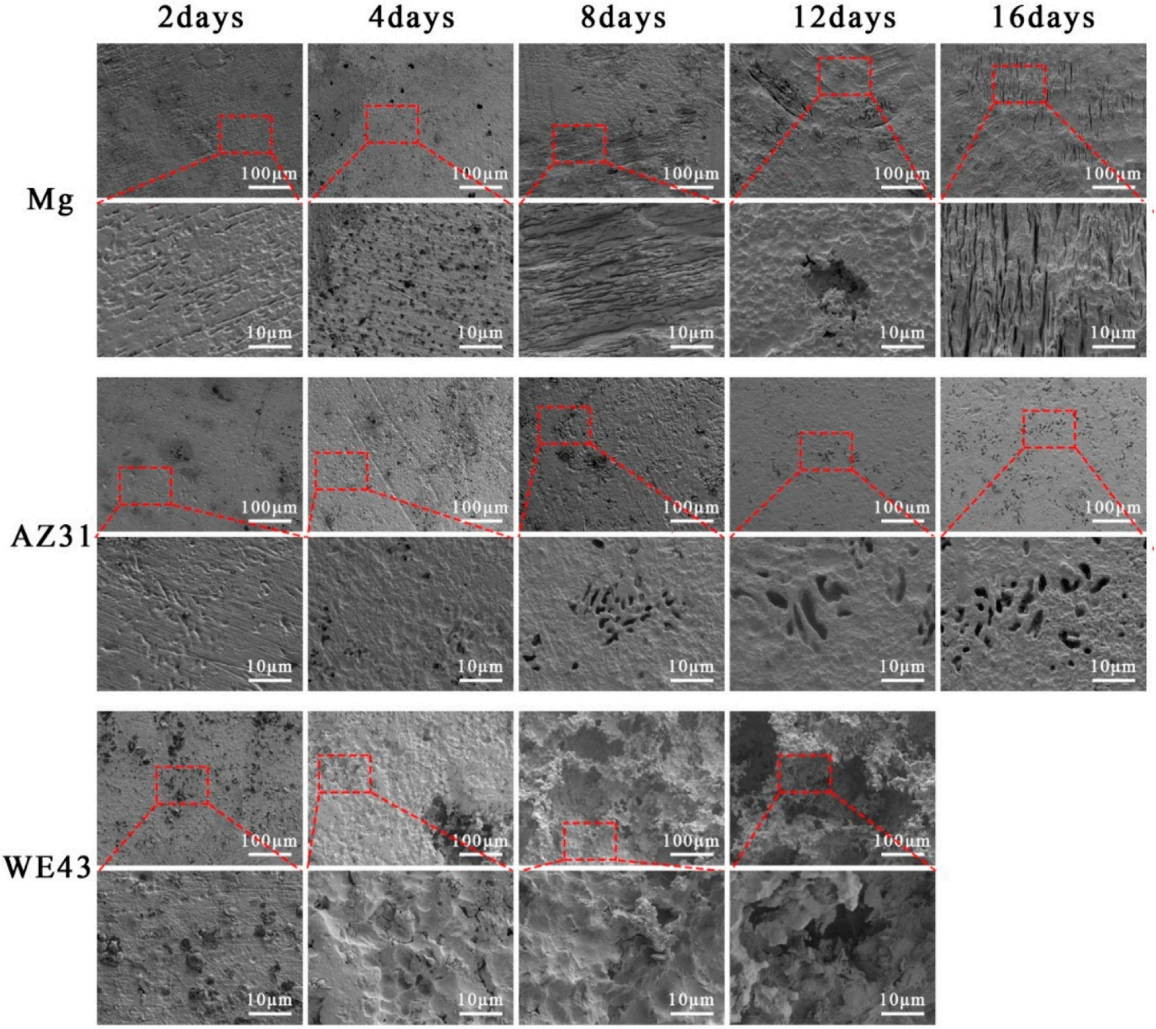
SEM of surface morphology of Mg, AZ31, and WE43 after static immersion in Hank’s solution at 37 ± 0.5°C (the second, fourth, and sixth line indicates the higher magnification of SEM pictures).

After the corrosion products on the sample surface were cleaned, the exposed material substrate is shown in Figure 4. The surface morphology of the pure Mg changed from the flat surface on the 2nd day, to the hole defect on the 4th day, and to the stripe morphology on the 8th day, and then the stripe-like morphology changed to wider cracks. The surface changes of AZ31 were the least during the whole 16-day corrosion period, and the corrosion morphology was a small hole. With the corrosion progress of WE43, the pits gradually grew larger, accompanied by local corrosion shedding of the substrate, resulting in further increase of pits.

**FIGURE 5:**
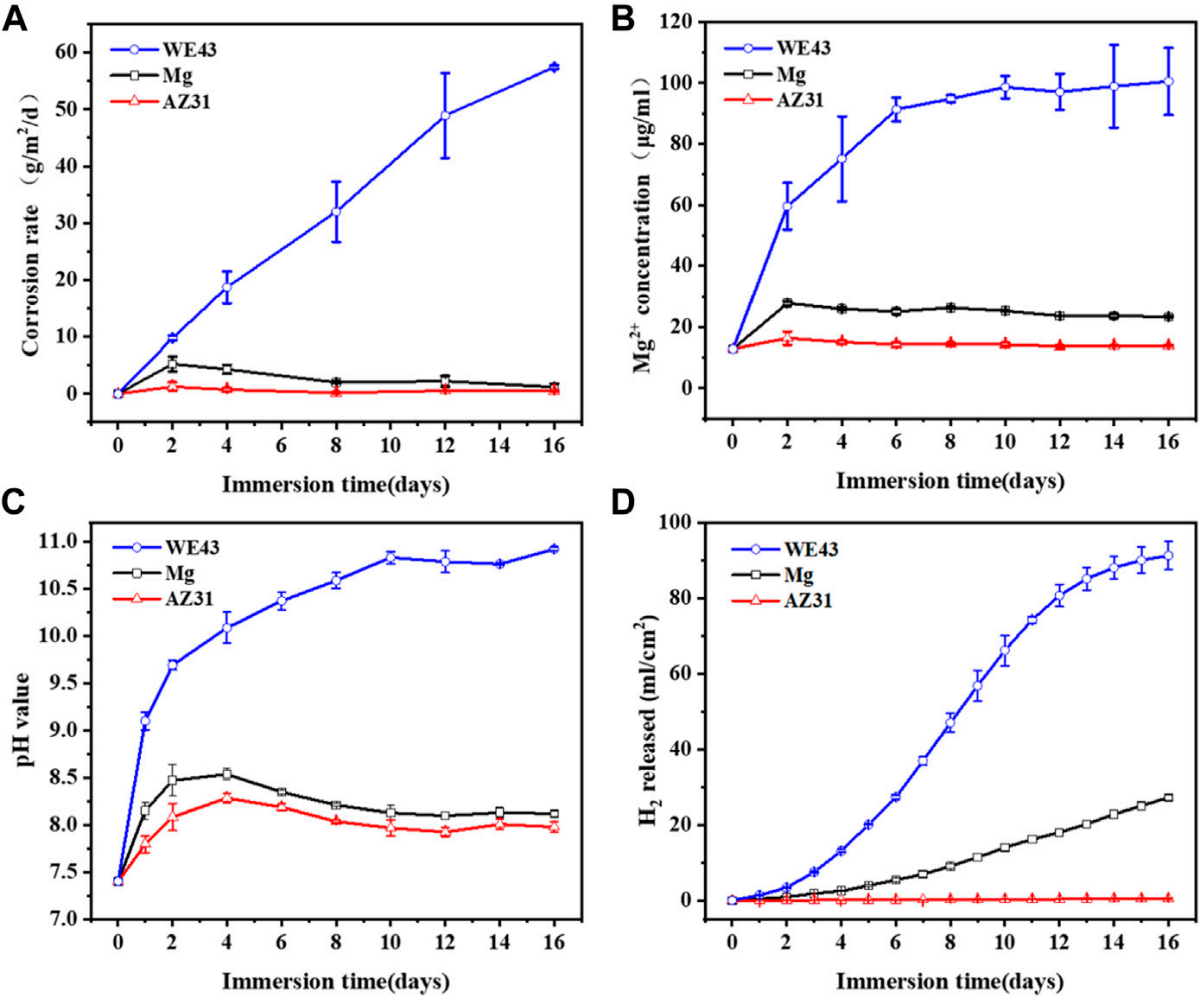
The corrosion rate **(A)**, Mg^2+^ concentration **(B)**, pH value **(C),** and H_2_ evolution **(D)** of Mg, AZ31, and WE43 after 16 days of static immersion in Hank’s solution at 37 ± 0.5°C.

As shown in Figure 5A, the order of corrosion rates is WE43 > Mg > AZ31. The corrosion rates of Mg and AZ31 had similar trends, which were different from that of WE43. The corrosion rate of WE43 increased almost linearly until the samples were completely corroded on the 16th day, and there were great differences between the parallel samples, which indicates that WE43 has poor corrosion resistance and unstable performance. However, the corrosion rates of Mg and AZ31 kept increasing in the first four days, and then decreased, and finally tended to be stable. The pH and Mg^2+^ concentration results of all the samples (Figures 5B,C) showed the same trend as corrosion rate. Because fresh Hank’s solution is changed every 2 days, when the corrosion rate after the refresh is lower than before, the Mg^2+^ concentration and pH at each time point will decrease compared with the previous time point (but still higher than the initial value). According to the cumulative amount of hydrogen released in Figure 5D, after 16 days of static immersion corrosion, the order of hydrogen evolution is WE43 (91.30 ± 3.76 ml/cm^2^) > Mg (27.23 ± 0.81 ml/cm^2^) > AZ31 (0.48 ± 0.07 ml/cm^2^). In the cumulative release of hydrogen (Figure 5D), the previous static corrosion rule of WE43 was more obvious since there was no replacement of fresh solution. The Mg samples showed a constant speed tendency of hydrogen evolution. AZ31 has almost no hydrogen evolution and shows excellent corrosion resistance.

### Electrochemical Corrosion Evaluation

**FIGURE 6:**
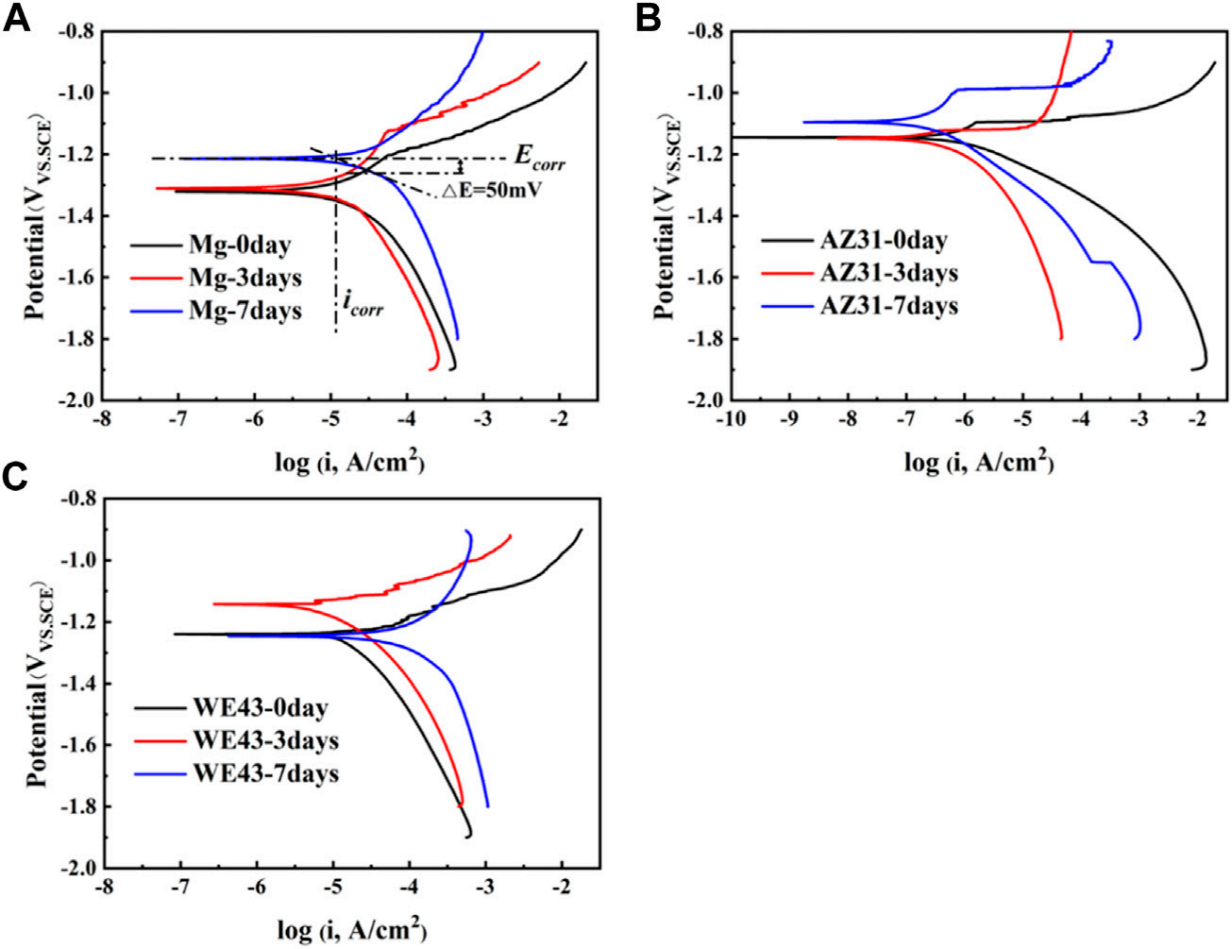
Polarization test results of Mg **(A)**, AZ31 **(B),** and WE43 **(C)** immersed in Hank’s solution (37 ± 0.5°C).

In order to further understand the corrosion behaviors of the Mg-based materials after static immersion, electrochemical tests were used to investigate the corrosion mechanism. The PDP results of the Mg-based materials immersed in Hank’s solution are shown in Figure 6. The E_corr_ and i_corr_ were determined *via* Tafel extrapolation (Figure 6A). The relevant data are shown in Table 1. The corrosion current density i_corr_ (mA/cm^2^) is related to the average corrosion rate P_i_ (mm/y) (ASTM, 2009) as shown in the following equation:

$${\text{P}}_{\text{i}}=22.85\;{\text{i}}_{\text{corr}}.$$

**TABLE 1:** Corrosion potentials E_corr_, corrosion current densities i_corr_, and the corrosion rate (P_i_) of WE43, AZ31, and pure Mg samples immersed in Hank’s solution at 37 ± 0.5°C.

	Immersion time (d)	E_corr_ (V)	Log (i_corr_, A/cm^2^)	P_i_ (mm/y)
Mg	0	−1.24	−5.55	0.064
	3	−1.31	−5.42	0.087
	7	−1.21	−4.92	0.274
AZ31	0	−1.15	−6.13	0.017
	3	−1.15	−6.63	0.005
	7	−1.10	−6.78	0.004
WE43	0	−1.24	−4.98	0.239
	3	−1.14	−5.41	0.089
	7	−1.25	**-**4.32	1.091

Smaller i_corr_ means better corrosion resistance of the sample (Shi and Atrens, 2011). According to Table 1, it can be roughly judged that corrosion resistance of Mg became worse with time, which of AZ31 is the opposite. The corrosion resistance of WE43 got better after three days of immersion and then turned worse after seven days of immersion. Moreover, the i_corr_ value of AZ31 is 1-2 orders of magnitude lower than that of Mg and WE43, showing better corrosion resistance. The i_corr_ value of WE43 is the highest, meaning the worst corrosion resistance, which is consistent with the results of static immersion test (Figure 5).

**FIGURE 7:**
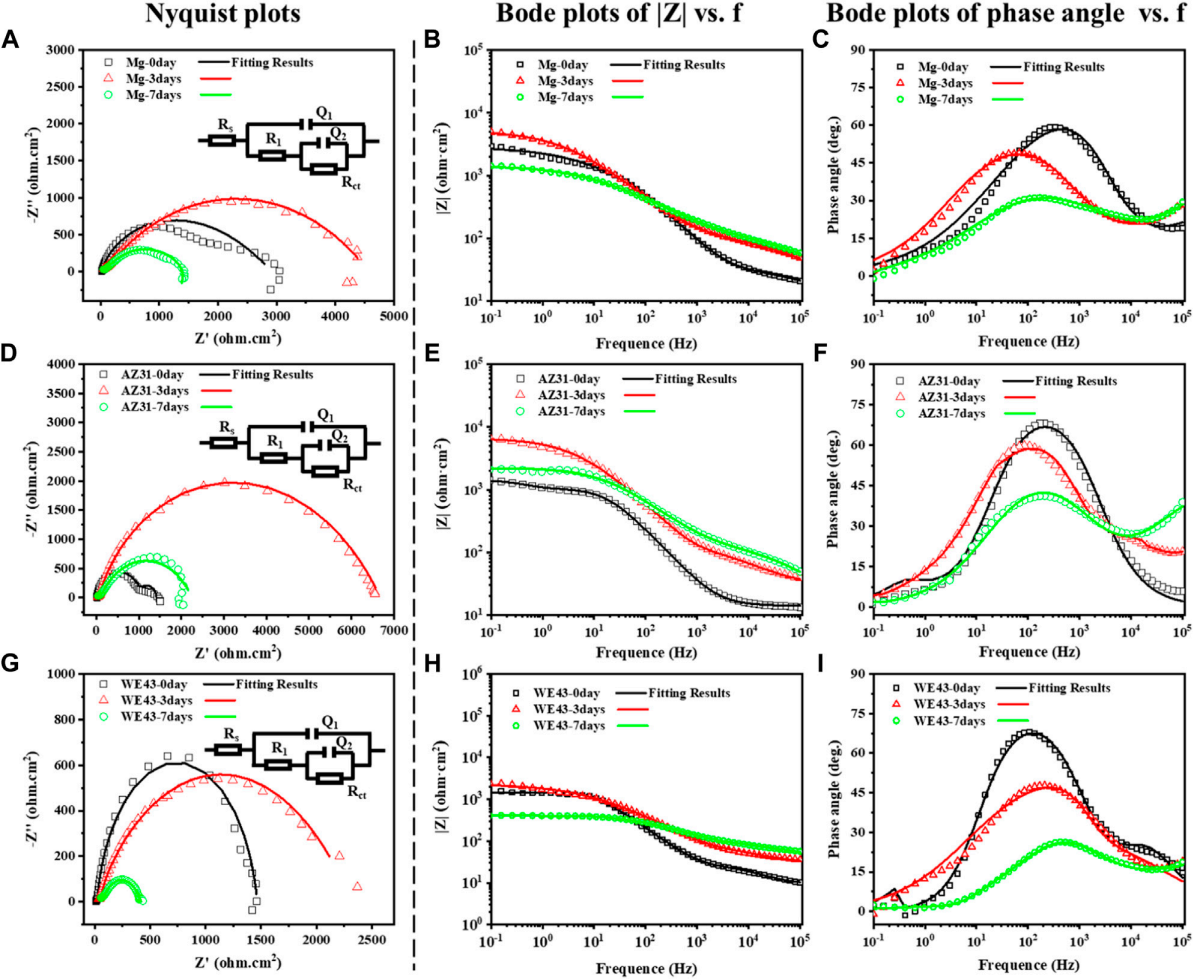
Nyquist plots and corresponding electrical equivalent circuit diagrams (EEC), Bode plots of |Z|, and phase angle vs. frequency of Mg **(A–C)**, AZ31 **(D–F),** and WE43 **(G–I)** immersed in Hank’s solution (37 ± 0.5°C).

EIS test results of the Mg-based materials immersed in Hank’s solution are shown in Figure 7, including Nyquist diagram and Bode diagram as well as their fitting plots. The radius of capacitive ring can reflect the speed of corrosion rate, and the larger radius of impedance represents better corrosion resistance (Song and Atrens, 2003). The radiuses of capacitive ring of all the Mg-based materials increase on 3d, and then reduce on 7d, indicating that the deposition of corrosion product layer on the sample surface can improve corrosion resistance in the initial stage, and then the protection of the corrosion product layer decreases as the corrosion progresses. The electrical equivalent circuit R_s_ {Q_1_ [R_1_ (Q_2_R_ct_)]} (Figure 7) can be proposed as the electrical equivalent circuit model to simulate the EIS results. In this model, R_s_ represents the solution resistance; Q_1_ and R_1_, respectively refer to the capacitance and resistance of surface corrosion layer; Q_2_ and R_ct_ relate to the double layer capacitance and resistance associated with charge transfer reaction, respectively. Corresponding fitted results are shown in Table 2. It can be discovered that the value of Rs is similar, indicating the validity of simulated results. The R_1_ of Mg exposed to the solution increases with time during the whole corrosion period, indicating the deposition of corrosion product layer on the sample surface. The R_1_ of AZ31 increases with the immersion time up to 3d, and it basically stays the same to 7d, indicating the stabilization of corrosion product layer on the sample surface due to less corrosion. In the case of WE43 samples, R_1_ values increase with the immersion time up to 3d, while an obvious reduction of R_1_ can be inspected afterward. This is ascribed to the local breakdown of the corrosion, which may be caused by the surface corrosion products falling off shown in Figure 3. The reaction resistance R_ct_ can be roughly correlated to the corrosion reaction resistance. Accordingly, the R_ct_ values of Mg and AZ31 at 7d are about 4–7 times larger than that of WE43, showing better corrosion resistance.

**TABLE 2:** Representative fitting results of EIS of Mg, AZ31, and WE43 samples immersed in Hank’s solution at 37 ± 0.5°C.

Samples	R_s_ (Ωcm^2^)	Q_1_ (s^n^Ω^−1^cm^−2^)	n_1_	R_1_ (Ωcm^2^)	Q_2_ (s^n^Ω^−1^cm^−2^)	n_2_	R_ct_ (Ωcm^2^)
Mg-0d	10.0	5.12E-05	0.46	37.7	3.52E-06	0.89	2873.4
Mg-3d	11.1	7.12E-07	0.73	80.6	4.19E-05	0.52	4473.0
Mg-7d	9.3	8.79E-05	0.40	252.3	1.17E-05	0.72	1251.0
AZ31-0d	14.1	1.43E-05	0.88	47.0	1.24E-03	0.97	1352.4
AZ31-3d	27.7	1.84E-05	0.62	133.7	1.73E-06	0.94	6522.0
AZ31-7d	4.8	3.21E-06	0.64	124.0	1.56E-05	0.68	2113.0
WE43-0d	9.3	4.58E-06	0.85	19.0	1.07E-05	0.91	1437.0
WE43-3d	27.3	5.86E-05	0.56	130.8	6.63E-07	0.99	2156.0
WE43-7d	16.7	8.32E-06	0.55	68.0	3.38E-05	0.66	332.0

### Dynamic Corrosion Evaluation

**FIGURE 8:**
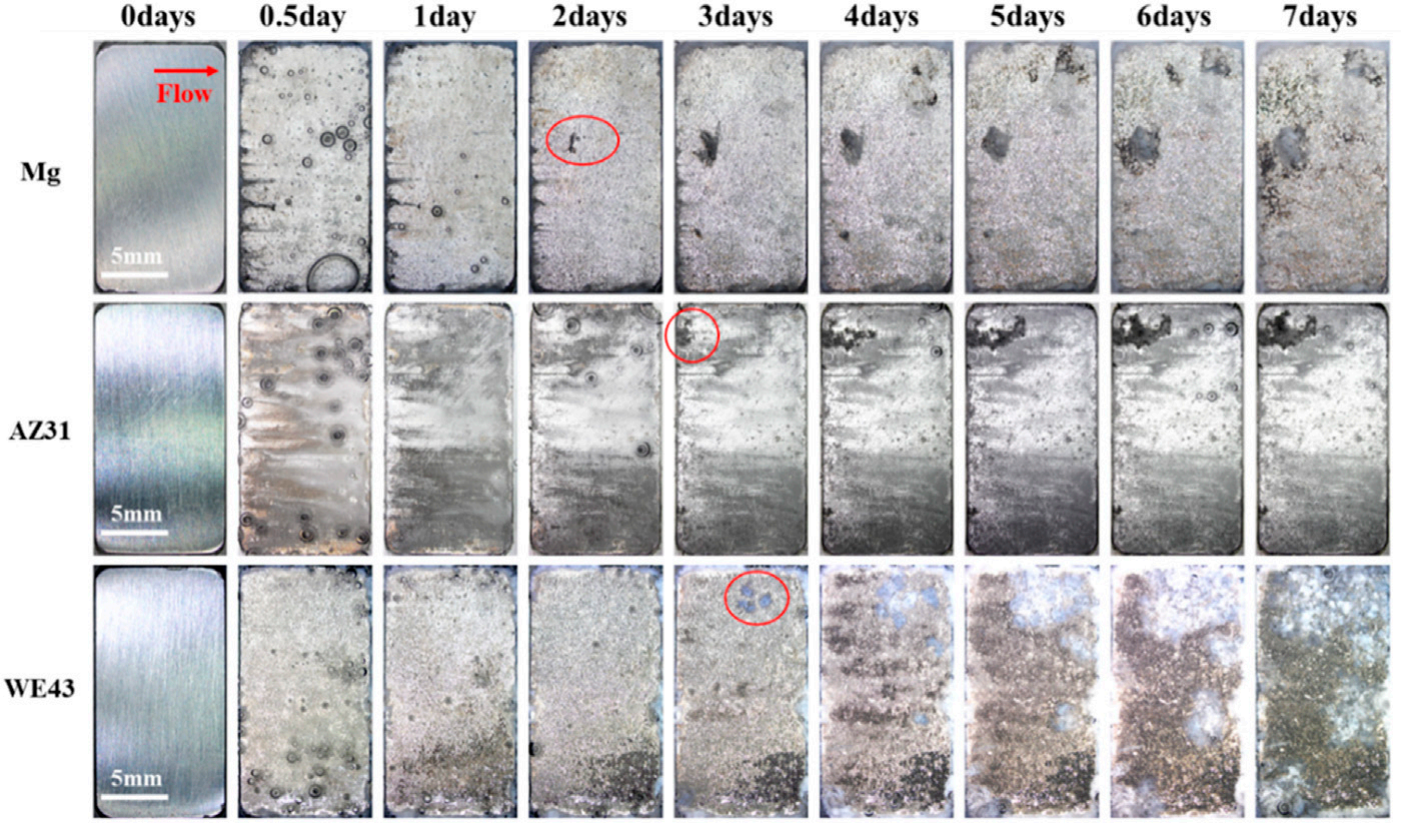
*In-situ* morphological images of dynamic corrosion of Mg, AZ31, and WE43 in flow chamber (with Hank’s solution at 37 ± 0.5°C). Red arrow indicates the flow direction. Red marked areas indicate the corrosion pits.

In order to intuitively study the degradation behaviors of the Mg-based materials under the action of near-physiological fluid shear stress, the corrosion morphologies of the samples were recorded at different time points in the flow chamber, as shown in Figure 8. After seven days of dynamic corrosion, a small number of corrosion products were observed on the surface of pure Mg group, no obvious corrosion products were deposited on the surface of AZ31, while a mass of corrosion products were deposited on the surface of the WE43 group. During the 7-day dynamic degradation process, most areas of the sample surface showed uniform corrosion. On the other hand, local corrosion pits appeared on the surface of all the samples, and the corrosion pit areas became larger with time. Obvious local corrosion pit appeared on the surface of pure Mg from the 2nd day, and grew to ∼6.25 mm^2^ on the 7th day. Meanwhile, more corrosion products also appeared in the corrosion pits. Local corrosion pit began to appear on the surface of AZ31 on the 3rd day and grew to ∼6.27 mm^2^ on the 7th day. On the surface of WE43, corrosion pits with corrosion products could be observed on the 3rd day and grew to ∼48.85 mm^2^ on the 7th day.

**FIGURE 9:**
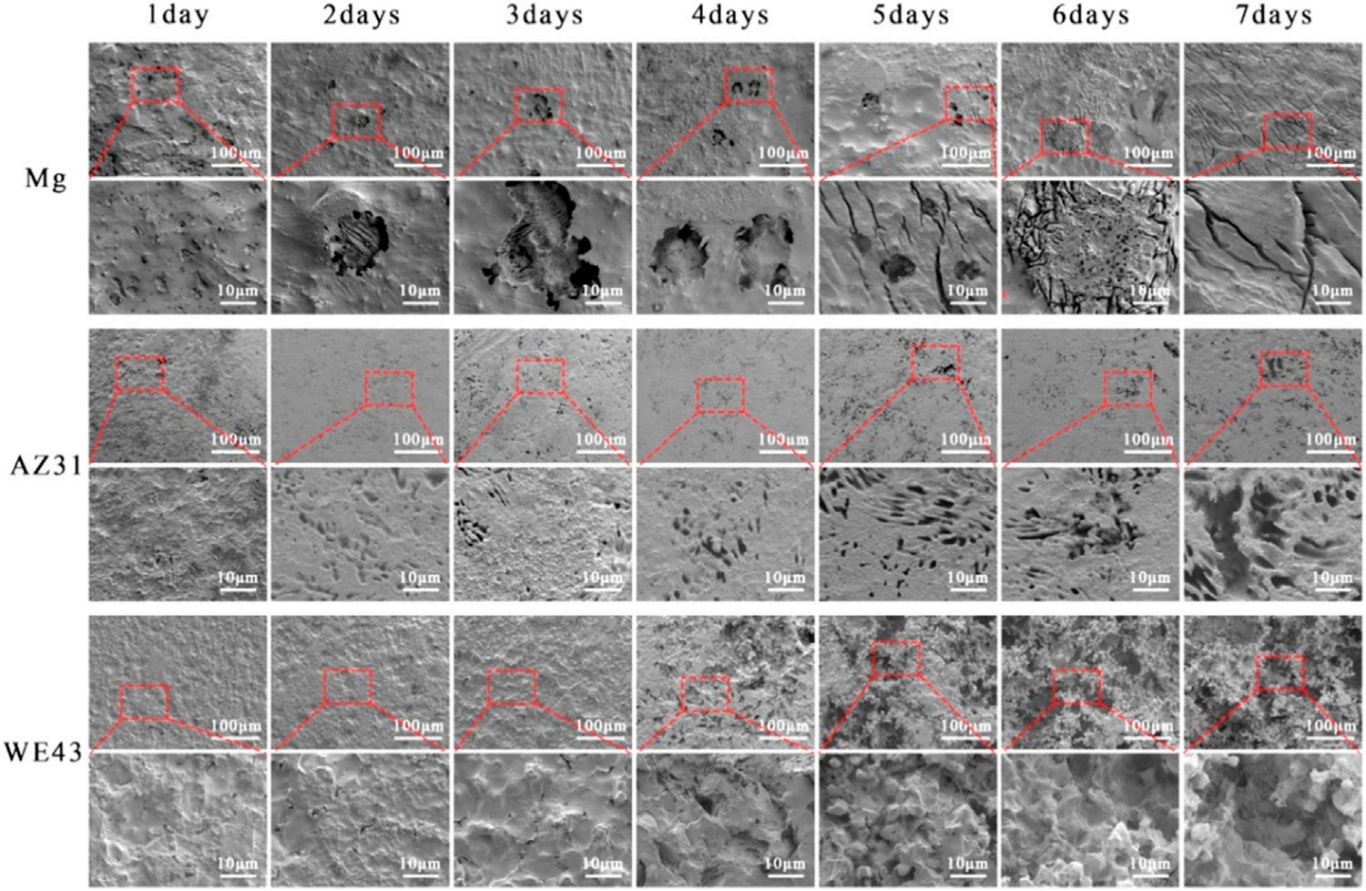
SEM images of the surface morphology of Mg, AZ31, and WE43 after dynamic immersion in Hank’s solution at 37 ± 0.5°C (the second, fourth, and sixth line indicates the higher magnification of SEM pictures).

After seven days of dynamic corrosion in the plate flow chamber, SEM images of Mg, AZ31, and WE43 corroded substrates are shown in Figure 9. As can be seen from the low multiple pictures, the surface morphologies of pure Mg and AZ31 were relatively flat during the dynamic degradation process of seven days. While the surface morphology of WE43 remained flat until the 3rd day, from the 4th day, obvious corrosion holes appeared on the surface, and developed into serious corrosion pits with time. As can be seen from the high multiple pictures, the surface of Mg remained with no defects on the 1st day, corrosion holes appeared from the 2nd day to the 4th day, and corrosion grooves appeared from the 5th day and became more and more serious with the corrosion process. The surface of AZ31 remained with no defects until the 2nd day, and corrosion holes began to appear from the 3rd day, and the corrosion holes became larger with the corrosion process. The changes of high multiples and low multiples of WE43 surface morphologies are consistent.

**FIGURE 10:**
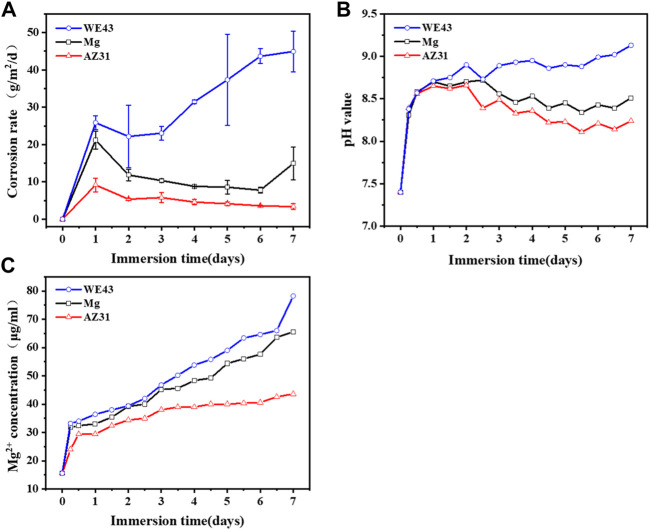
Corrosion rate **(A)**, pH value **(B),** and Mg^2+^ concentration **(C)** of Mg, AZ31, and WE43 in a dynamic flow chamber with Hank’s solution at 37 ± 0.5°C.

As shown in Figure 10A, the corrosion rate of AZ31 reached the maximum on the 1st day (9.17 ± 1.84 g/m^2^/d), and then decreased continuously until it reached 3.36 ± 0.76 g/m^2^/d on the 7th day. The corrosion rate of Mg reached the maximum value on the 1st day (about 21.17 ± 2.36 g/m^2^/d), then continued to decrease to 7.75 ± 0.80 g/m^2^/d on the 6th day, but increased again on the 7th day, reaching 14.93 ± 4.35 g/m^2^/d. The corrosion rate of WE43 reached 25.83 ± 1.89 g/m^2^/d on the 1st day, then decreased briefly to 23.06 ± 1.85 g/m^2^/d on the 3rd day, and then increased continuously to 44.93 ± 5.46 g/m^2^/d on the 7th day. Correspondingly, the solution pH of AZ31 and Mg rapidly reached the maximum value of about 8.7 on the 1st day, and continued to decrease until the 7th day, and reached about 8.2 and 8.4, respectively. However, the pH of WE43 rapidly reached about 8.7 on the 1st day, and then rose slowly until it reached about 9.1 on the 7th day. The Mg^2+^ concentration is presented in Figure 10C. In general, WE43 and Mg releases Mg^2+^ more rapidly with time than AZ31. On the 7th day, Mg^2+^ concentration of WE43 and Mg in the circulating solution reaches 78 μg/ml and 68 μg/ml, respectively, which is about 1.5 times higher than that of AZ31 (43 μg/ml).

The results in Figure 10 suggest that the rapid corrosion of the Mg-based materials on the 1st day leads to a rapid increase in pH and magnesium ion concentration. As the active magnesium on the surface of the material was replaced by magnesium oxide and corrosion products, the corrosion rate decreased and the pH and magnesium ion concentration increase rate slows down. Due to the shear stress of the fluid, the corrosion products were washed away and the damage of the substrate, by the aggressive ions in the medium, was accelerated, which resulted in the increase of corrosion rate of WE43 and Mg again. Correspondingly, AZ31 showed the best corrosion resistance.

Dynamic corrosion after seven days was compared with static corrosion after eight days, the corrosion rate of WE43 in dynamic corrosion is 1.4 times higher than that in static corrosion. The corrosion rate of Mg (14.93 ± 4.35 g/m^2^/d) is seven times higher than that in static corrosion (2.00 ± 0.20 g/m^2^/d), and the corrosion rate of AZ31 (3.36 ± 0.76 g/m^2^/d) is 20 times higher than that (0.17 ± 0.15 g/m^2^/d) in static corrosion. These results show that the dynamic conditions evidently accelerate the corrosion rate.

### Growth Behavior of HUVECs and HUASMCs

**FIGURE 11:**
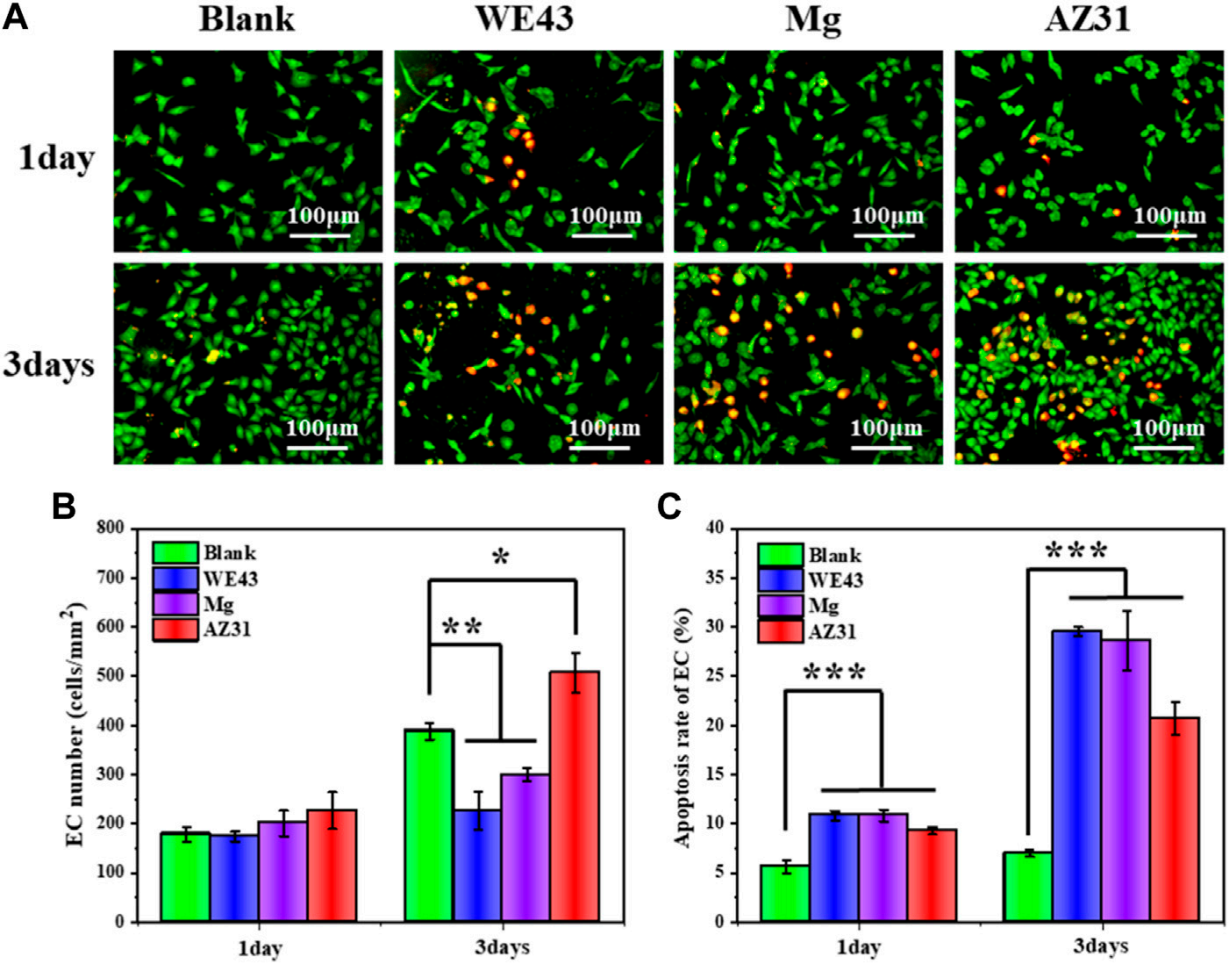
**(A)** Representative fluorescence images of HUVECs adhered on the well plates after culture with each group of samples for 1 and 3 days. **(B)** Statistical counting of HUVECs on the well plates. **(C)** Apoptosis rate of HUVECs (data presented as mean ± SD and analyzed by ANOVA, *p < 0.05, **p < 0.01, ***p < 0.001).

The fluorescence images of ECs (Figure 11) showed that the cells maintained a fusiform appearance, and the number of ECs cultured in the extracts for one day was not significantly different from that of the blank control group, revealed nontoxic effect of the extracts. After three days of culture, the number of ECs in the WE43 and Mg group was significantly lower than that in the blank group, indicating that the two groups of extracts had a certain inhibitory effect on the proliferation of ECs, while the number of cells in the AZ31 group was significantly higher than that in the blank group, indicating that the extract of AZ31 group could promote the proliferation of ECs.

On the other hand, after one day of culture, the apoptosis rate of ECs in the three groups of extracts was about 5% higher than that in the blank group, and after 3 days of culture, the apoptosis rates of the WE43 group and Mg group were both about 20% higher than that in the blank group, and the AZ31 group was also about 13% higher than that in the blank group, indicating that all the extracts had side effects on ECs. Generally, high pH would inhibit ECs growth (Cutaia et al., 2005). According to the abovementioned degradation behavior analysis, the AZ31 group had the lowest corrosion rate and the lowest pH change, while the pH of other groups changed greatly, which may be the main reason for the inhibition of cell growth of other groups. In addition, magnesium ions have been reported to promote endothelial cell proliferation (Zhao and Zhu, 2015). As the AZ31 group was less negatively affected by pH, the proliferation of endothelial cells was promoted by combining the positive effects of magnesium ions.

**FIGURE 12:**
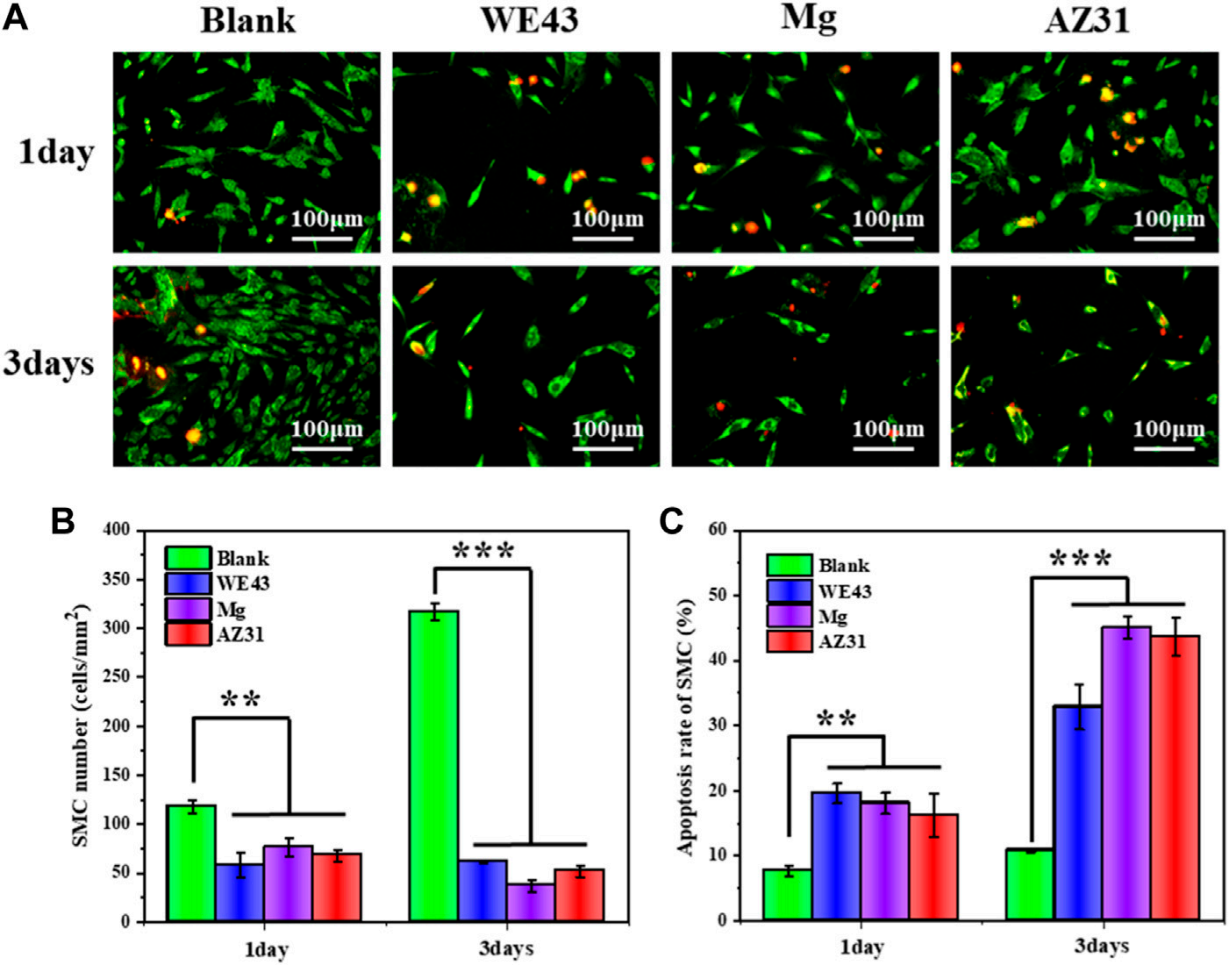
**(A)** Representative fluorescence images of HUASMCs adhered on the well plates after culture with each group of samples for one and three days. **(B)** Statistical counting of HUASMCs on the well plates. **(C)** Apoptosis rate of HUASMCs (data presented as the mean ± SD and analyzed by ANOVA, *p < 0.05, **p < 0.01, ***p < 0.001).

As shown in Figure 12, the number of SMCs cultured in the extracts for one day was significantly reduced compared with the blank control group. After three days of culture, the number of cells in the extract group was significantly less than that in the blank group, indicating that the three groups of extracts had a significant inhibitory effect on the proliferation of SMCs. Moreover, the results of cell apoptosis rate were consistent with them. After three days of culture, the cell apoptosis rate of the WE43 group reached 32.8%, Mg group and AZ31 group even reached 45.1 and 43.6%, respectively, far higher than that of the blank group (10.6%), indicating that the extracts had great side effects on SMCs. The growth behaviors of SMCs in the Mg-based materials group were mainly inhibited. This may be related to the pH value and magnesium ion concentration of the extracts. The degradation of the Mg-based materials resulted in the increase of pH value and magnesium ion concentration of the extract. On one hand, higher pH inhibits cell growth. On the other hand, magnesium ion has been reported to inhibit the proliferation of smooth muscle cells by regulating gene expression and to promote the proliferation of endothelial cells with minor effects on gene expression at the same concentration (Sternberg et al., 2012).

By comparing the growth behavior of ECs and SMCs cultured in the extracts for three days, ECs had different degrees of proliferation, while SMCs did not proliferate, indicating that the Mg-based material extracts were relatively friendly to ECs, but had a significant inhibitory effect on SMCs. This effect is beneficial to re-endothelialization of stent implantation site and inhibition of SMCs hyperplasia. In particular, the vascular cellular responses of AZ31 indicated a potential application for stents. Moreover, Erişen et al. reported that the dissolved Al ions during the degradation of AZ31B alloy did not cause a danger for the viability of the nerve cells in the *in vitro* and *in vivo* studies (Erişen et al., 2022). This, once again, shows that biodegradable AZ31B magnesium alloy stent has a prospect of application.

## Conclusion

In this study, we investigated the *in vitro* degradation behaviors of pure Mg, AZ31, and WE43 under the action of shear stress close to the WSS of the human coronary artery, and compared them to that of the samples immersed in static solution. The results revealed that both in static and dynamic conditions, the order of corrosion rates is WE43 > Mg > AZ31. This indicates that the corrosion rate of different Mg-based materials is related to the composition of the elements. The dynamic conditions evidently accelerate the corrosion rate. After seven days of testing, the corrosion rates of WE43, Mg, and AZ31 in dynamic corrosion is 1.4 times, 7 times, and 20 times higher than that in static corrosion, respectively. The extracts of AZ31 samples were relatively friendly to ECs, but significantly inhibited the growth of SMCs. In conclusion, AZ31 shows excellent corrosion resistance and suitable effect on vascular cell, which is worthy of further study as a potential vascular stent material.

## Data Availability

The original contributions presented in the study are included in the article/Supplementary Material. Further inquiries can be directed to the corresponding authors.
